# Comparison of Stiffness Measurements of Wooden Rods Using Acoustic Guided Wave and Static Bending Test Techniques

**DOI:** 10.3390/s25164930

**Published:** 2025-08-09

**Authors:** Adli Hasan Abu Bakar, Mathew Legg, Khalid Mahmood Arif, Daniel Konings, Fakhrul Alam

**Affiliations:** 1 Colleges of Sciences, Massey University, Auckland 6330, New Zealand; adlihasan6315@gmail.com (A.H.A.B.); k.arif@massey.ac.nz (K.M.A.); 2 Department of Mechanical Engineering, National University of Singapore, Singapore 117575, Singapore; 3 School of Engineering, New Zealand Skills and Education College, Auckland 1010, New Zealand; 4 Department of Mechanical Engineering, Auckland University of Technology, Auckland 1010, New Zealand; daniel.konings@aut.ac.nz; 5 Department of Electrical & Electronic Engineering, Auckland University of Technology, Auckland 1010, New Zealand; fakhrul.alam@aut.ac.nz

**Keywords:** radiata pine, rod velocity, rod, ToF, guided waves, MoE

## Abstract

Traditionally, mechanical bending tests are used to measure the stiffness of lumber, which is generally represented by the static modulus of elasticity (MoE). However, it is desirable to measure the stiffness of wood before it is processed into lumber. Acoustic nondestructive testing techniques are therefore the main techniques used by the wood industry to estimate the dynamic MoE of wood. The acoustic resonance technique is employed for measuring the MoE in felled logs and lumber. In contrast, the acoustic time-of-flight (ToF) technique is traditionally used for MoE measurements on standing trees and seedlings. However, the ToF technique overestimates stiffness compared to both resonance and static bending tests (considered the gold standard). In this work, a guided wave technique is used to measure the stiffness of wooden rods. This work is the first to compare the MoE values obtained using static bending tests (gold standard) with those obtained using acoustic resonance, ToF, and guided wave methods. Measurements were performed on 16 mm diameter radiata pine wooden rods. For comparison, measurements were also performed on acetal, aluminium, and steel rods of similar dimensions. The findings show that stiffness measurements obtained using the proposed guided wave method are more accurate than those obtained using the traditional ToF method and closely match those of the resonance method across all materials. The measurements from the ToF method were overestimated compared to resonance, guided wave, and static bending methods. The findings show the potential for the guided wave method to be used as an alternative method to provide more accurate stiffness measurements in juvenile trees/seedlings compared with the traditional ToF technique.

## 1. Introduction

The ability to measure wood properties is important for the wood industry. Stiffness is a key property of wood since it determines if timber can be used for structural applications. The gold standard method for measuring the stiffness of wood is to use static bending tests. Stiffness measurements obtained from this technique are often referred to as the static modulus of elasticity (MoE). The technique involves applying a load onto a sample at a constant rate and measuring the resulting deflection. The MoE is then obtained from the resulting load–deflection curve. Static bending tests are generally only performed after the wood has been processed into lumber. Timber that is found to be below the threshold required for structural grade might then be discarded. Not only is this wasteful, but it also means that the wood cannot be used for another application it might have been more suited to. This can reduce profits and reduce the sustainability of the wood industry [1]. Additionally, static bending tests are generally not suited for field testing due to the size and weight of the equipment.

A range of nondestructive testing (NDT) techniques have therefore been developed to assess wood stiffness before it is processed into an end product. Examples are SilviScan [2], X-ray tomography, Near-Infrared (NIR) spectroscopy, and acoustic techniques [3]. Acoustics is the most popular NDT technique for measuring wood stiffness due to its affordability and simplicity [4]. Increased efficiency, sustainability, and profitability can be achieved by the wood industry using acoustic NDT techniques [5,6]. For example, acoustic NDT techniques can be used for pre-sorting of logs prior to milling to allow sawmills to process logs with higher stiffness levels into structural timber and process lower-stiffness logs into other end products [7]. Acoustic NDT techniques can also be used to estimate the stiffness of standing trees for applications such as the valuation of forests. Additionally, acoustic NDT methods are also used on seedlings and juvenile trees in breeding trials to improve the stiffness levels of future plantation forests [8,9].

### 1.1. Relationship Between Stiffness and the Speed of Sound in Wood

The stiffness of wood obtained using acoustic NDT techniques is commonly referred to as the dynamic MoE. Wood is classified as an orthotropic and anisotropic material, which means its acoustic velocity and mechanical properties are different in three directions—longitudinal, tangential, and radial. This variation in the speed of sound in wood with direction depends on the orthotropic mechanical properties of the wood (three Young’s moduli, three shear moduli, and six Poisson’s ratios).

The stiffness of wood correlates directly with the MoE along the longitudinal axis and is generally assumed to be

(1) $$E_{L}=\rho \;{c_{l}}^{2},$$

where $E_{L}$ is the dynamic MoE along the longitudinal axis, ρ is the density of the material, and $c_{l}$ is the acoustic velocity propagating in the longitudinal direction. However, it should be noted that Equation (1) is an approximation. It does not include other factors that influence the speed of sound in wood, such as the moisture content, temperature, and the presence of knots. Also, this equation assumes wave propagation in an isotropic and homogeneous thin rod. However, in reality, wood is generally orthotropic, inhomogeneous, and has a finite diameter. If an acoustic signal propagates a sufficient distance along an elongated rod of wood, it will propagate as guided waves where different wave modes propagate at different speeds [10].

### 1.2. Acoustic Resonance and Time of Flight Testing

The stiffness of logs and timber samples can be estimated using the acoustic resonance NDT method. In this technique, longitudinal stress waves are induced by striking one end of the sample with a hammer in the direction of the grain. A device such as a transducer, microphone, or accelerometer is then used to measure the received signal at one of the ends of the sample. The harmonic resonance frequencies are then identified using a Fast Fourier Transform (FFT). The resonance velocity $c_{res}$ is then obtained using

(2) $$c_{res}=\frac{2Lf_{n}}{n},$$

where *L* is the length of the sample, $f_{n}$ is the *n*th harmonic frequency, and *n* is an integer (*n* = 1, 2, 3…). In practice, the first or second harmonic is commonly used in resonance measurements of wood.

A limitation of the resonance technique is that it cannot be used on standing trees or seedlings, as it requires cut ends. Instead, a technique called the time-of-flight (ToF) method is used for this application. The ToF method typically employs two transducers that are normally mounted on probes. The probes are driven into the timber sample, and a hammer strikes one of the probes to induce stress waves. The ToF velocity $c_{tof}$ can be obtained using

(3) $$c_{tof}=\frac{\Delta d}{T},$$

where Δd is the spacing between the two probes and *T* denotes the time taken for the stress waves to propagate from one probe to the other. Typically, the ToF method uses the First Time of Arrival (FToA) of the signal at each receiver. This time is usually obtained by determining when the acoustic signal captured by the receiver first goes above a threshold value.

Studies have shown good correlations between MoE values obtained using both acoustic resonance and ToF methods with those obtained using static bending tests [11,12,13]. However, the ToF method systematically overestimates stiffness values compared to the static bending and resonance methods [10,14]. This correlates with the ToF method having measured speeds of sounds that are consistently higher than those obtained with the resonance technique by an amount ranging from 7 to 35% [10,14]. This overestimation of the ToF technique has been reported to vary depending on a number of factors, such as the species, age, and diameter of the tree stem [10,14].

Potential causes of the overestimation have been suggested. These include the variation in stiffness from the core to the outer layers [15] and the difference in the propagation distance of the acoustic signals and hence the types of waves (bulk waves versus rod waves) that are used for ToF and resonance techniques [16]. In reference [17], the authors proposed that dispersion might contribute to the ToF velocities’ overestimation of stiffness. Dispersion is the phenomenon where different frequency components of an acoustic signal propagate at different speeds. This causes the signal to distort and spread out as it propagates. The authors suggested that this dispersion could cause distortion of the leading edge of the signal during propagation, and this might cause errors when calculating the FToA for the signal using the threshold method. The authors proposed a dispersion compensation technique to address this issue for the ToF measurements. However, this required some knowledge of the dispersion in the sample and testing was only performed on thin rods.

In summary, the ToF technique has been the only acoustic technique available for measuring the stiffness of standing trees and seedlings. However, it has limitations in terms of accuracy. It systematically overestimates stiffness measurements. This overestimation varies significantly and varies depending on a range of factors. It is desirable to develop an alternative acoustic technique that could be used for standing trees and seedlings. Acoustic guided wave testing is proposed here as a potential alternative method to ToF.

### 1.3. Guided Wave Acoustic Testing

An acoustic signal will propagate in an unbounded or infinite medium as bulk waves, where all frequencies propagate at the same speed. However, if the medium has geometric boundaries (e.g., cylinder or plate), the acoustic signal will propagate as guided waves composed of different types of vibrations called wave modes. These wave modes are usually dispersive, meaning that different frequencies propagate at different speeds. In plate-like structures, the guided waves generated are referred to as Lamb waves. In contrast, for cylindrical rod-like structures, different types of guided waves propagate, which are referred to as rod waves.

Ultrasonic guided waves have been used for nondestructive testing of a wide range of structures. They have traditionally been mainly used for metal structures. For example, ultrasonic guided waves are commonly used for the detection of corrosion in pipelines for the oil and gas industry [18]. There have been a few studies that have used guided wave techniques for nondestructive testing of wood, though this area of study is still in its early stages. Guided waves have been used to detect rot and insect damage in timber utility poles and to estimate how deep they are embedded into the ground [19,20]. There have also been a few studies that have utilised Lamb guided wave techniques to measure the elastic constants for rectangular wood samples [21,22]. A few of these have compared the estimated mechanical properties of rectangular wooden plates with those obtained using static bending methods [23,24,25].

In our previous work [26], a new NDT technique was developed to obtain the dynamic MoE of wooden rods by using multi-frequency guided wave measurements to obtain a “rod velocity’’. This velocity was compared with the acoustic velocities obtained through traditional resonance and ToF methods. The velocity of the proposed guided wave method was close to that of resonance. In contrast, the ToF technique gave velocity measurements that were significantly higher than those of resonance. However, a limitation of that research was that it did not include static bending tests. Additionally, that study only performed measurements on two wooden rod samples. It was therefore difficult to evaluate how accurate the new guided wave technique was at estimating stiffness.

In this article, we build upon that previous research. Resonance velocity, ToF velocity, and guided wave rod velocity measurements are performed on a range of wooden, steel, aluminium, and acrylic rod samples. The velocities for each sample are used to calculate dynamic MoE values for the resonance, ToF, and guided wave techniques. These are then compared with MoE values obtained using three-point static bending tests.

This study is the first work to compare the dynamic MoE values obtained from guided wave, resonance, and ToF techniques with those obtained using static bending tests. It is indeed the first work to compare mechanical bending test MoE values with guided wave dynamic MoE values for wooden cylindrical rod samples. The results showed that the guided wave technique gives dynamic MoE values comparable to resonance, with significantly lower overestimation relative to static bending tests compared to the traditional ToF method. The results indicate that guided wave testing has the potential to be used as an alternative method for measuring the stiffness of seedlings and juvenile trees with reduced errors compared with the traditional ToF technique.

This paper is structured as follows. The methodology and experimental procedure are described in Section 2. The experimental results are discussed in Section 3. Finally, the discussion and conclusion with recommendations for future work are, respectively, presented in Section 4 and Section 5.

## 2. Materials and Methods

The objective of this study is to compare dynamic MoE measurements obtained using resonance, ToF, and guided wave methods with static MoE obtained from static bending tests. To achieve this, fifteen cylindrical radiata pine (*Pinus radiata D.Don*) rods were purchased commercially. These rods are undamaged, defect-free, and have a diameter of 16 mm and a length 2440 mm. These samples were purchased from a timber supplier. Therefore, they are likely to have come from different trees and different locations within each tree. The samples might include wood from different anatomical regions, such as heartwood and sapwood, which can further contribute to variability in their properties. As a result, the wood properties such as density and stiffness may vary between samples.

It was decided that the same MoE measurements should also be performed using rods of different materials, where the wave propagation is easier to model and the mechanical properties are known. Specifically, tests were performed on five samples of each material: Aluminium 6061-T6, Stainless Steel 304, and acetal (polyoxymethylene-copolymer). These have similar dimensions to the wood samples that were tested. These materials were chosen since they have significantly different MoE values. The aluminium and steel rods have diameters of 16 mm and the acetal rods have diameters of 16.6 mm.

**Figure 1:**
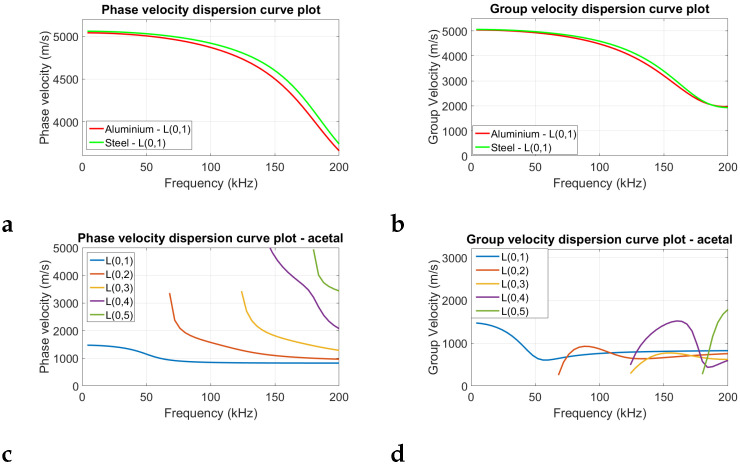
Theoretical dispersion curves generated using GUIGUW. Plots (**a**,**b**), respectively, show the phase and group velocity dispersion curves for aluminium and steel. Plots (**c**,**d**) show the phase and group velocity dispersion curves for acetal.

Figure 1 presents the theoretical dispersion curves of the longitudinal wave mode for the aluminium, steel, and acetal samples depicted in the group velocity–frequency domain, which were obtained using GUIGUW [27]. The theoretical plots were obtained using the mechanical properties shown in Table 1. These values were taken from a mid-range of commonly cited mechanical properties for these materials. The plots show that for aluminium and steel, only the fundamental longitudinal wave mode L(0,1) is present in the frequency range of 0–200 kHz. Meanwhile for acetal, up to the fifth-order longitudinal wave mode can be present. Additionally, the longitudinal wave modes for acetal are more dispersive (higher rate of change in group velocity with change in frequency) than those for the steel and aluminium samples.

Acoustic resonance, time-of-flight, guided wave, and static bending tests were performed on all of the rod samples. The experimental setup used for each of these measurement techniques is described in the following sections.

The density of the samples was calculated according to the ASTM D2395 standard [31]. A MD918 moisture meter was used to measure the moisture content of the wooden samples.

### 2.1. Resonance

**Figure 2:**
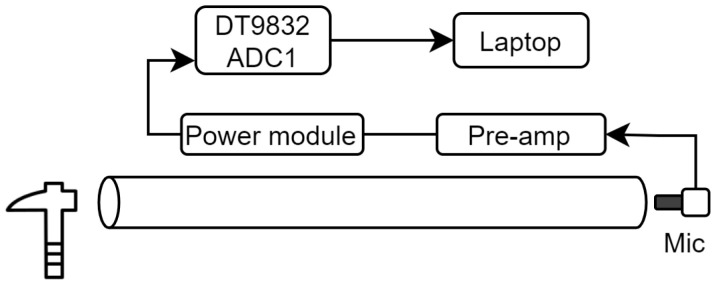
Diagram of the experimental setup for resonance measurements.

Resonance measurements were obtained by tapping a hammer at the sample front to induce longitudinal stress waves. The vibrations were measured using a GRAS (Holte, Denmark) 46BF-1 microphone that was positioned close to the other end of the sample. The microphone captures frequencies ranging from 4 Hz to 100 kHz through a GRAS 26A-1 pre-amp. This was powered by a GRAS 12AK module. The ADC channel from a Data Translation (Marlborough, MA, USA) DT9832 DAQ module was used to sample this signal. In this study, a sampling rate of 2 MHz was used for all measurements. The data was saved to file and analysed using MATLAB (version 2023B). The experimental setup is shown in Figure 2.

**Figure 3:**
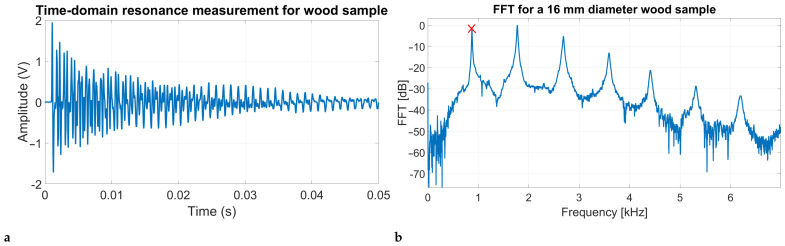
Plots showing how the resonance speed is calculated for a 16 mm diameter wooden sample. Plot (**a**) shows an example time-domain signal captured for a resonance speed measurement. Plot (**b**) shows the frequency-domain version of this signal with the fundamental frequency $f_{1}$ peak marked with a red “×” symbol.

The received signal was processed using a Fast Fourier Transform (FFT) to identify the resonance peak frequencies $f_{n}$, refer to Figure 3b. The resonance velocity $c_{res}$ was then obtained by averaging the acoustic velocities obtained for the first and second harmonics, which were calculated using Equation (2). The resonance dynamic MoE was then calculated using Equation (1) by substituting the value of $c_{res}$ into $c_{l}$.

### 2.2. Time of Flight

**Figure 4:**
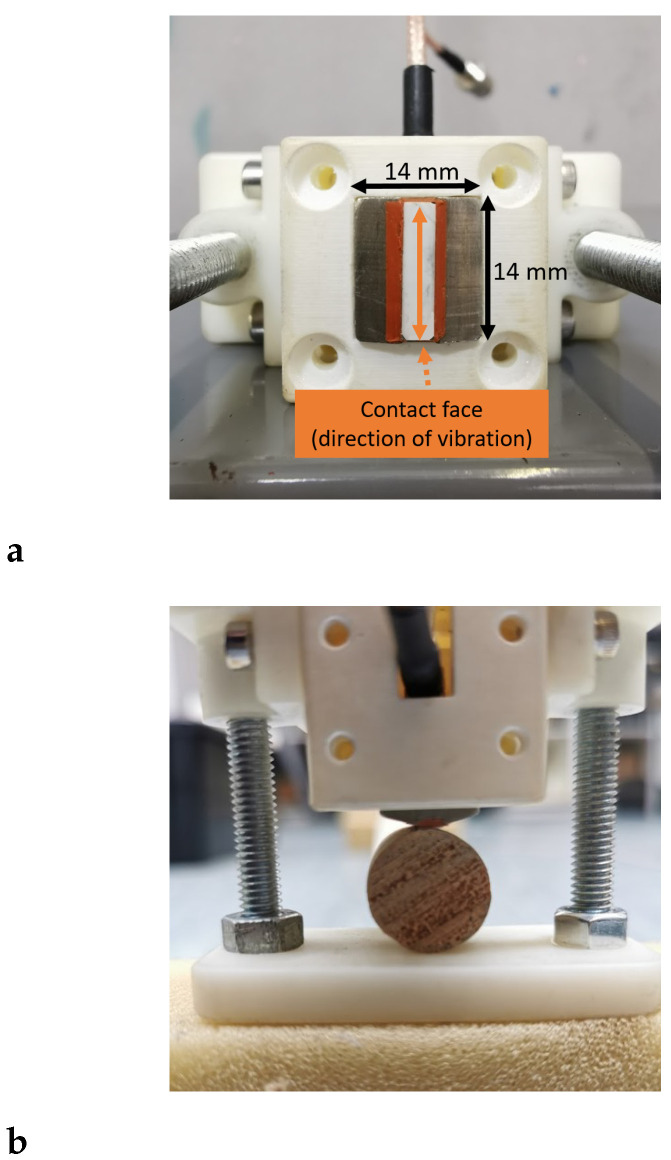
Photo (**a**) shows the shear transducer used in this study (with dimensions). Photo (**b**) shows the transducer being clamped onto a wooden sample using springs for tension.

Dry-coupled shear PZT transducers were used in this study for excitation/reception of vibrations [32,33], see Figure 4a. The transducers have a broad frequency range with a consistent flat frequency response across the desired frequencies. The receivers were clamped using springs to provide dry coupling to the sample, see Figure 4b. Additionally, the receivers were aligned along the grain direction to amplify the reception of vibrations in the longitudinal directions.

**Figure 5:**
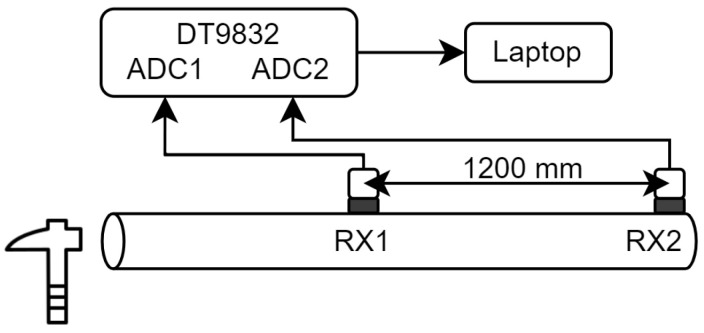
Diagram of the experimental setup for ToF measurements.

For ToF measurements, two transducers acting as receivers were used and were positioned as shown in Figure 5. Longitudinal vibrations were generated using a hammer hit in the direction along the grain at the opposite end of the sample. The signal recording commenced with a keystroke just before the hammer was struck. Similar to the resonance method, a sampling frequency of 2 MHz was used and the received signal was sampled using ADC channels from the DT9832 module. The data was stored as a MAT-file an analysed using MATLAB.

**Figure 6:**
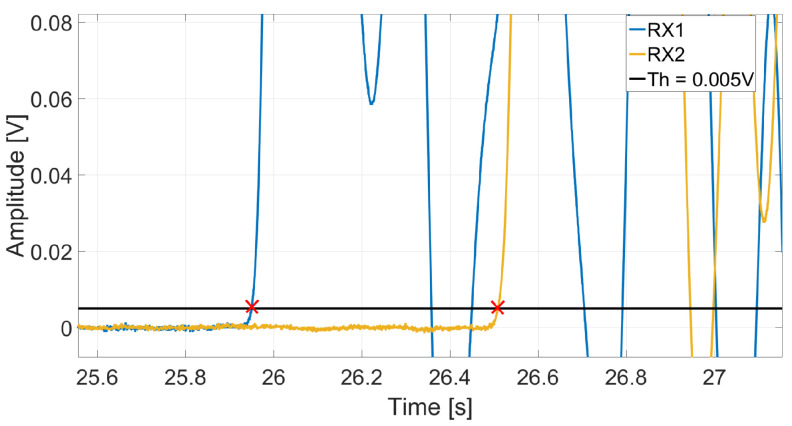
Example plot showing how a threshold value was used to estimate the propagation time for ToF velocity measurements. The red crosses show where the signal measured by each receiver transducer first gooes over the threshold value.

The FToA of the received signals was determined using the amplitude threshold method, where a fixed threshold value was applied, see Figure 6. This threshold, set at five standard deviations of the noise, was carefully chosen to avoid false positives. The average of 10 measurements per sample was taken as the average ToF velocity. This ToF velocity was then used in Equation (1) to obtain the ToF dynamic MoE. Note that in this work, the dispersion compensation technique described in reference [17] has not been used to correct ToF measurements, as we wanted to compare results with the traditional method.

### 2.3. Guided Waves

**Figure 7:**
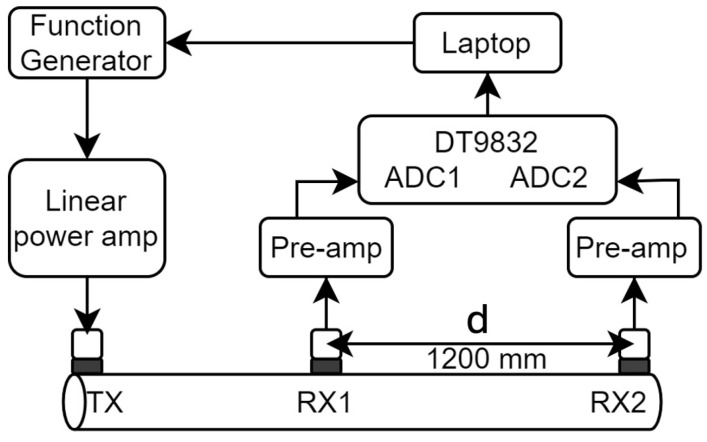
Diagram of the experimental setup for guided wave measurements.

Guided wave measurements were conducted using three transducers arranged in a pitch–catch configuration. The configuration can be seen in Figure 7. The excitation signal was generated using MATLAB. For excitation, 5 cycles of a sine wave ranging from 15 to 40 kHz in 1 kHz increments were used. A Hanning window was applied to the signal for narrowband excitation. The signal was generated from an Agilent (Santa Clara, CA, USA) 33220A function generator and subsequently amplified to 400 Vpp using a custom-built linear power amplifier. The receiver transducer signals were amplified using custom-designed pre-amps, and the received signal was sampled using the DT9832 DAQ module’s ADC channels with a sampling rate of 2 MHz. Synchronisation of the excitation and reception of the signal was obtained using hardware triggering.

The rod velocity $c_{o}$, which is the phase velocity of the L(0,1) wave mode as the frequency approaches zero, was then calculated. The technique used to obtain the rod velocity was developed by the authors and is presented in reference [26]. The process is described below. An initial guess is first made of what the L(0,1) phase velocity $v_{ph}$ dispersion curves might be. This could be a straight line or a curve obtained using an analytical approximation of the L(0,1) wave mode. The received signal $g_{1}$ at transducer RX1 is then transformed into the frequency domain using a Fast Fourier Transform (FFT) to give $G_{1}\left(\omega \right)$. The propagation of the signal over the distance *d* (the distance between the two receiver transducers) is then simulated using the frequency domain shifting

(4) $$Y\left(\omega \right)=G_{1}\left(\omega \right)\;e^{-j\left(\frac{\omega }{v_{ph}\left(\omega \right)}\right)d-\alpha \left(\omega \right)d},$$

where α(ω) is an assumed attenuation curve of the L(0,1) wave mode. This simulated propagation allows for dispersion and attenuation. A time-domain version y(t) of this artificially propagated signal is then obtained by taking the inverse Fourier transform of Y(ω). The Root Mean Squared Error (RMSE) between the artificially propagated signals and the actual signal $g_{2}\left(t\right)$ measured by transducer RX2 is then calculated using

(5) $$RMSE=\sqrt{\frac{\sum {\left[g_{2}\left(t\right)-y\left(t\right)\right]}^{2}}{N}},$$

where *N* is the number of samples. An iterative process is then used where the phase velocity dispersion curve is incrementally adjusted (in 1 m/s steps) and the attenuation curve is adjusted until a minimum RMSE value is achieved. The phase dispersion curve that gives the lowest RMSE value is selected and the phase velocity value at the central transmit frequency is selected and used as the actual phase velocity of the sample at this frequency.

The procedure described above was repeated for the transmit frequencies, which ranged from 15 kHz to 40 kHz in 1 kHz intervals. A curve was then fitted through the resulting 25 phase velocity measurements, as is shown in Figure 9 of reference [26]. This curve was obtained using

(6) $$c_{l}=c_{o}\sqrt{\frac{1+a_{1}\;a_{2}\;k^{2}}{1+a_{1}\;k^{2}}},$$

where $c_{o}$ is the rod velocity, $a_{1}$ and $a_{2}$ are coefficients, and *k* is the wave number. This is based on a correction for the Rayleigh–Bishop theory [34]. Least squares fitting was used to obtain the rod velocity $c_{o}$ as well as the parameters $a_{1}$ and $a_{2}$. This “rod velocity” will be termed the guided wave velocity hereafter. The guided wave dynamic modulus of elasticity (MoE) was subsequently computed using this velocity in Equation (1).

### 2.4. Static Bending Test

**Figure 8:**
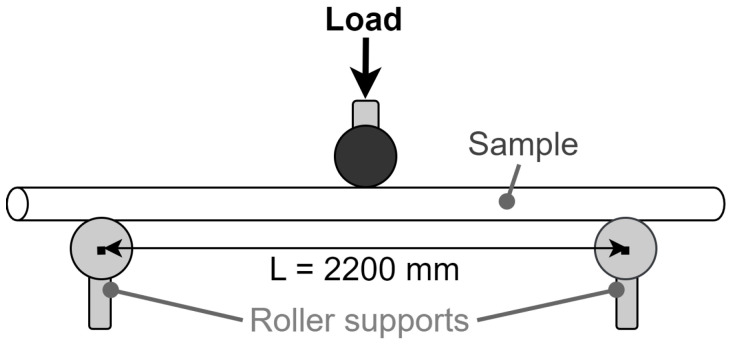
Diagram of a three-point static bending test with centre load.

**Figure 9:**
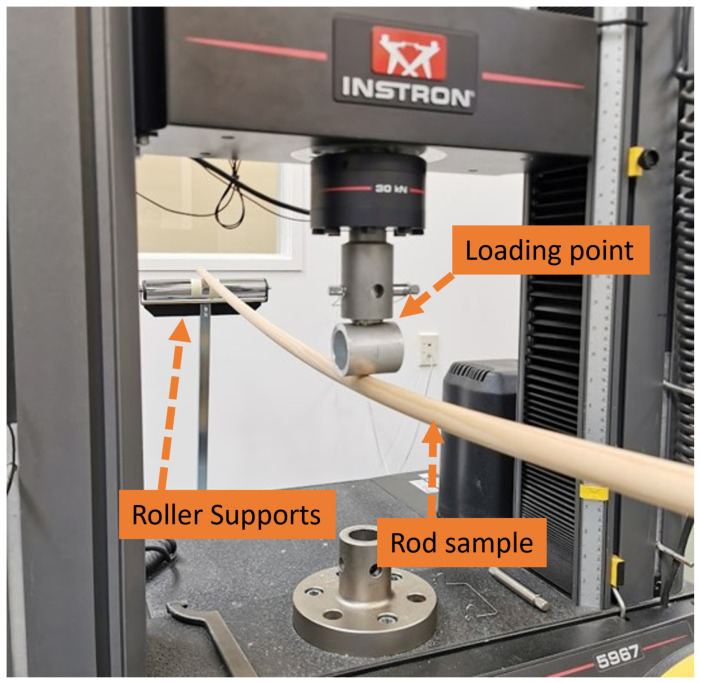
Experimental setup for the three-point bending test using the Instron 5967 Universal Testing Machine.

For comparison, static MoE measurements of the samples were obtained using three-point bending tests. The tests were performed using an Instron 5967 Universal Testing Machine according to the ASTM D198 standard [35]. Roller supports were positioned near the edges of the sample for support and a span length of 2200 mm was used, see Figure 8. A photo of the experimental setup for the three-point bending test on one of the wooden samples is shown in Figure 9.

Load was applied to the centre of the sample at a loading rate of 15 mm/min. The three-point bending test was performed until an elongation of 100 mm at the midpoint was achieved. The displacement of the sample was measured using the Instron machine’s built-in displacement sensor. The static MoE was then calculated using

(7) $$E_{S}=\frac{L^{3}}{48\;I}\frac{F}{\Delta x},$$

where *F* is the load (N), *L* is the span length (mm), Δx is the displacement at midspan (mm), and *I* is the moment of inertia (mm^4^). Since the samples are rods of circular cross-section, *I* is given by

(8) $$I=\frac{1}{4}\pi r^{4},$$

where *r* is the radius of the rod in mm.

## 3. Results

### 3.1. Dynamic and Static MoE Measurements

Table 2 shows the density, acoustic velocity, dynamic MoE, and static MoE values obtained for the fifteen radiata pine cylindrical rod samples. The moisture content of the samples is 8.5%, with no variation between samples. The measured density of the samples ranges between 395 and 655 kg/m^3^, as seen in Table 2. Note that the variation in density is expected as the wood samples may be taken from different parts of a tree or different trees. The average density of the samples is approximately 525 kg/m^3^, with a standard deviation of 68 kg/m^3^. The minimum and maximum values of static MoE obtained were 8.00 and 17.00 GPa, respectively, whereas the dynamic MoE values ranged from 8.31 to 19.33 GPa. The density and stiffness values obtained in this study are within the range reported in the literature for radiata pine [36].

**Figure 10:**
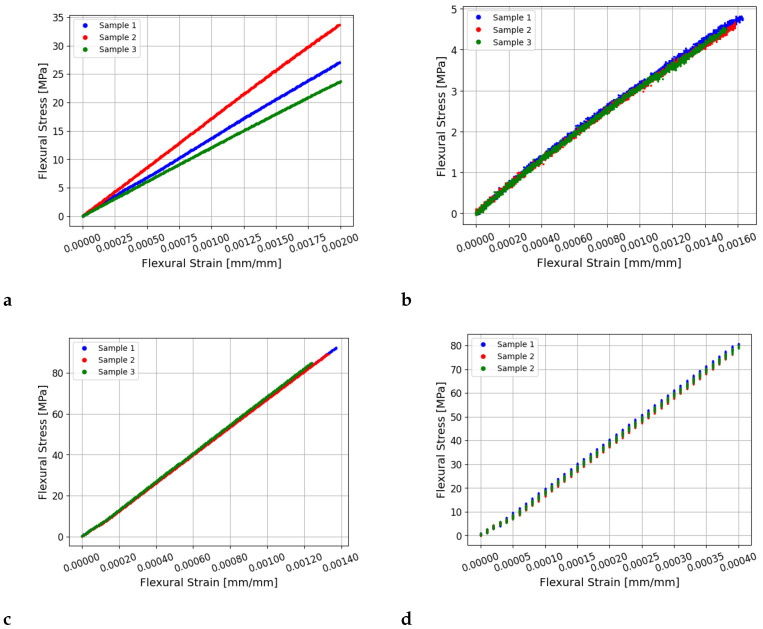
Plots of the flexural strain versus stress measurements obtained during static bending tests for the (**a**) wood, (**b**) acetal, (**c**) aluminium, and (**d**) steel samples. Measurements from three samples are shown for each material tested.

Figure 10 shows the measured flexural strain versus stress measurements for the four materials. The measurements for all 15 wooden samples are shown. However, for the acetal, aluminium, and steel samples, measurements are only plotted for three of the five samples tested since the measurements for these materials overlapped. The measurements appear to show some slight slipping of the sample on the rollers when the load was first applied, particularly for the steel and aluminium samples.

Table 3 provides the averaged measured MoE values for the wood, acetal, aluminium, and steel rod samples. For the wooden samples, the average was made over fifteen sets of measurements, while for the other materials, the average was made over five sets of measurements. The findings indicate that dynamic MoE measurements obtained via the ToF method were slightly overestimated for all materials compared to the MoE values derived from static bending tests.

### 3.2. Comparison Between Static and Dynamic MoE

#### 3.2.1. Wood MoE Comparison

**Figure 11:**
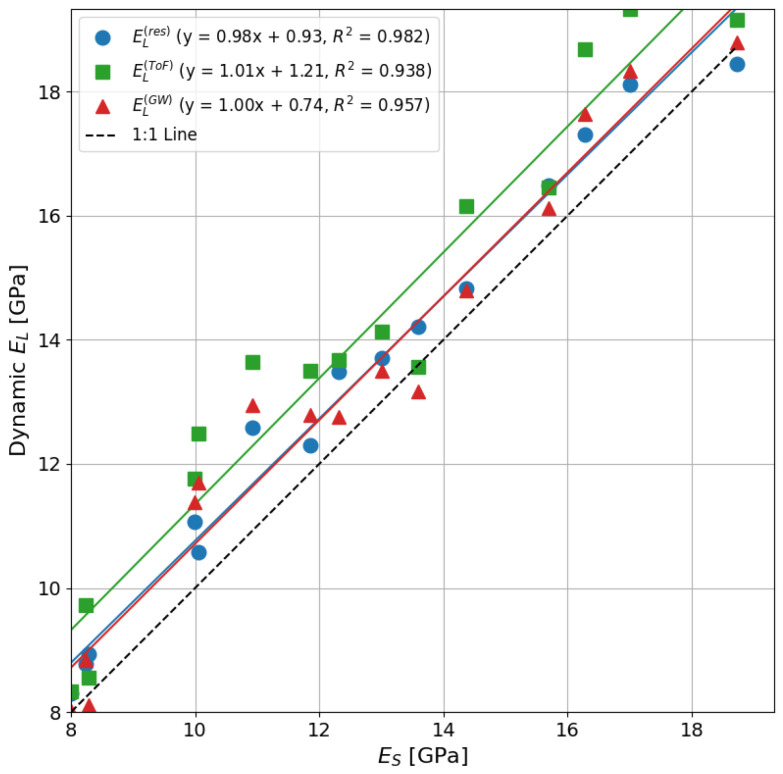
Relationship between dynamic and static MoE for 16 mm diameter wooden rod samples.

Figure 11 shows the relationship between dynamic MoE and static MoE values obtained for the wooden rod samples. The figure shows good agreement between dynamic MoE values obtained using resonance, ToF, and guided wave methods and static MoE values. The coefficient of determination, $R^{2}$ value, is used to measure the goodness of fit of the regression models to predict static MoE from dynamic MoE values. High $R^{2}$ values (0.93–0.98) were obtained between the dynamic MoE and static MoE values. The results show that dynamic MoE values obtained using resonance, ToF, and guided wave methods are higher compared to static MoE values.

An $R^{2}$ value of 0.98 was obtained using the resonance method for the wooden samples. Resonance dynamic MoE values were on average 6.4% higher compared to the static MoE values. Similar results were achieved by Lindstrom et al. [37], who obtained stiffness measurements using the resonance method on radiata pine samples. The authors obtained an $R^{2}$ value of 0.93, and the results from the study show that dynamic MoE values were on average 4–7% higher compared to static MoE. Previous studies on other wood species have also obtained resonance dynamic MoE values, which were approximately 5–10% higher compared to static MoE values [38,39]. The resonance dynamic MoE values obtained in this study are consistent with those reported in the literature. More work is needed to investigate the cause of the variation between the resonance dynamic MoE and static MoE values.

Using the ToF method, an $R^{2}$ value of 0.93 was obtained for the wooden samples. The ToF dynamic MoE values for wood were on average 11.6% higher than static MoE, which is a significant overestimation. This ToF overestimation is expected, as it has been reported in the literature for wood [10,14]. Studies have reported that ToF measurements can vary due to factors such as the ToF tool used [40], the distance between probes [41], the position of probes [42], and the signal strength [43]. The results of this study show relatively small variations in ToF measurements. This may be because small-diameter rod samples were used.

Guided wave dynamic MoE values were on average 6.6% higher than static MoE values for the wooden samples. These results are higher than those reported by Fathi et al. [23,24], who performed measurements on rectangular wooden plates. The difference may be due to the geometric shape of the samples, as cylindrical rods were used in this study. Furthermore, the authors estimated the elastic modulus by measuring the Lamb wave shear velocity and shear modulus. In this study, the longitudinal wave velocity is used to directly measure the elastic modulus. The guided wave method used in this study produced results that are close to those of the resonance method. This suggests that similar stress waves are being propagated and measured using both methods.

The ToF method produced acoustic velocity and stiffness measurements that were overestimated compared to resonance, guided wave, and static bending methods. The variation between the ToF and guided wave methods may be due to dispersion effects [17]. Dispersion can cause distortion of the ToF received signal, which can lead to higher ToF measurements. The difference between guided wave and ToF measurements may be larger for larger-diameter samples, as it is expected that higher dispersion will be observed in larger-diameter samples. However, more work is needed to investigate this.

#### 3.2.2. MoE Comparison for All of the Materials Tested

**Figure 12:**
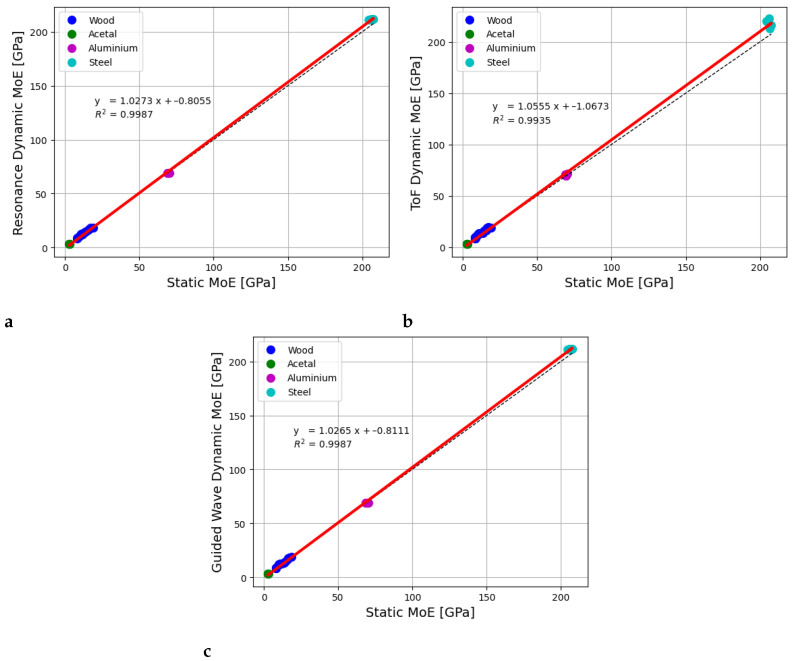
Relationship between MoE values obtained using the (**a**) resonance, (**b**) ToF, and (**c**) guided wave techniques compared to static bending tests for wood, acetal, aluminium, and steel rods.

The results presented in the previous section showed that the dynamic MoE values for the wooden samples were higher than the static MoE values obtained using static bending tests. This overestimation was higher for the ToF technique compared to resonance or guided waves. However, it was not clear if this was specific to wood or if similar results would also be obtained for rod samples made of other materials. Therefore, the tests were repeated with acetal, aluminium, and steel rods of similar dimensions to those of the wooden samples. Figure 12 shows plots of static MoE values versus dynamic MoE values obtained using resonance, ToF, and guided waves for all of the materials, including wood. All the techniques provided good fits between the static and dynamic MoE values. The resonance, ToF, and guided wave techniques all provided $R^{2}$ values of 0.99. However, the fitted lines throughout the data are higher than the 1:1 line (expressed as dashed lines) for each method. This shows that there is an overestimation in all of the dynamic MoE techniques over all four materials. This overestimation is higher for the ToF technique.

### 3.3. Relationship Between Dynamic MoE and Density for Wood

**Figure 13:**
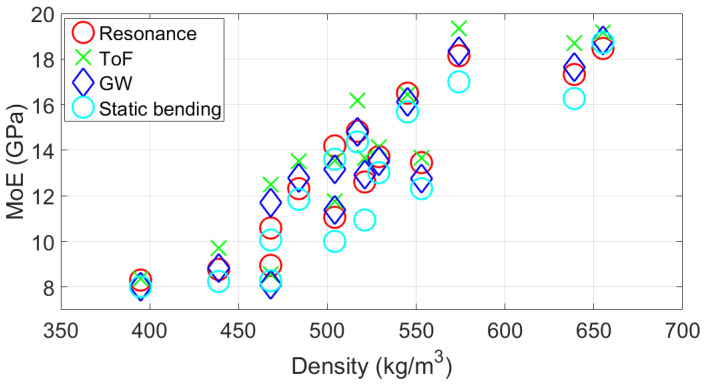
Relationship between MoE values obtained using different methods and density of the wooden samples.

Researchers have reported the importance of density in determining wood quality [44,45]. Figure 13 shows the relationship between dynamic and static MoE values as a function of density. The figure shows a positive linear relationship between the MoE values using all four methods with respect to density.

To quantitatively measure the linear relationship between the MoE and density, Pearson’s correlation coefficient (r-value) is used. A positive correlation coefficient indicates the tendency for one variable to increase or decrease together with another variable [46]. Table 4 shows the correlation values between density and the MoE values. The table shows high correlations (0.89–0.90) between the two variables. The correlation values obtained in this study are higher than those reported in the literature for radiata pine. Ivkovic et al. [2] and Lindstrom et al. [47] obtained correlation values ranging between 0.39 and 0.71 for radiata pine samples aged between 3 and 8 years old. The difference in correlation between MoE and density may be related to the growth rings and outerwood proportion, which could lead to biased results [37]. Previous studies on other tree species have reported strong correlations between density and dynamic MoE. For example, Illic [48] obtained correlations ranging from 0.81 to 0.83 between density and resonance dynamic MoE values. Chauhan and Sethy [49] obtained correlations ranging from 0.73 to 0.74 between density and dynamic MoE obtained using resonance and ToF methods for eight different wood species. Conversely, some studies have cautioned against relying solely on density to predict MoE [50,51] or have reported no correlation between them [52].

## 4. Discussion

In this work, dynamic MoE values obtained using the guided wave technique are compared with traditional resonance, ToF, and static bending methods for cylindrical wooden samples. The measurements were conducted on fifteen cylindrical radiata pine rods measuring 16 mm in diameter and 2440 mm in length. Tests were also performed on five samples each of acetal, aluminium, and steel rods of similar diameters and lengths to the wood samples. This was conducted as these materials had known mechanical properties and covered a range of MoE values.

For wood, the static MoE obtained in this study has a range of 8.00–18.73 GPa, whereas the dynamic MoE has a range between 8.02 and 19.33 GPa. Regressive models for MoE produced high $R^{2}$ values, which ranged from 0.93 to 0.98 using the resonance, ToF, and guided wave methods. Also, the wooden rod samples have a density range of 395–655 kg/m^3^, with an average density of 519 kg/m^3^. Strong correlations (r-value = 0.89–0.90) were observed between dynamic MoE and density, see Table 4. This is consistent with the results found in the literature, as discussed in Section 3.3.

The dynamic MoE values for all materials (wood, acetal, aluminium, and steel) were plotted against static bending test MoE values. This was performed for resonance, ToF, and guided waves. The dynamic MoE values obtained for each of these methods were fitted to the corresponding static MoE values and $R^{2}$ values of 0.99 were obtained. However, these plots show that there is a systematic overestimation of the measured dynamic MoE relative to static MoE. Table 5 provides the percentage overestimations of the measured dynamic MoE values relative to static MoE for each of the materials tested. The ToF technique gives the highest overestimations. The resonance and guided wave techniques produced slight overestimations (except for the aluminium samples) compared to static MoE values.

Table 5 shows that the MoE overestimation of the ToF method relative to static bending tests is different for different materials, with wood having the highest overestimation, followed by acetal. Could this be due to the differences in dispersion of the longitudinal wave modes for the different materials? In our previous study [17], it was shown that dispersion effects can cause a change in the rise time at the start of the signal. This difference in rise time could affect FToA measurements obtained using amplitude threshold, which can lead to an overestimation in the calculated wave speed. The L(0,1) wave mode phase velocity dispersion curve for a wooden rod, which had similar dimensions to those used in this work, was shown in our previous work to be significantly higher than that of an aluminium rod of similar dimensions [17]. Similarly, we can see from the dispersion curves shown in Figure 1 that acetal also has a higher dispersion of its longitudinal L(0,1) wave mode in the frequency range of 10–50 kHz compared to that of steel or aluminium and also has higher order wave modes appearing. Could this higher dispersion for wood and acetal (and potentially the presence of higher-order wave modes for acetal) be causing the higher overestimation for this material relative to steel and aluminium?

It is possible that the there were errors in the static bending test elastic modulus measurements. The three-point bending test assumes that the material behaves linearly elastically. That is to say that the stress is proportional to strain (Hooke’s Law) and deformation is fully recoverable and there is no shear deformation. For the case of the acetal in particular, this may not have been the case. There was some permanent deformation (bending) of these samples after the measurements. The technique also assumes that there is no slipping, friction, or noise due to the supporting heads. In actual fact, these may have been present. These factors may lead to an underestimation of the static modulus of elasticity measurements. Could an error like this account for why static bending tests had slightly lower values than resonance and guided wave methods?

## 5. Conclusions

The ability to measure wood properties, particularly stiffness, is important for the wood industry as it can have a significant effect on the profitability and efficiency of the wood industry. The acoustic resonance and time of flight techniques are the main nondestructive testing methods used to measure the stiffness of wood. However, resonance cannot be used for standing trees and juvenile trees/seedling. The time-of-flight method can be used for this application but systematically overestimates stiffness estimate values. This work investigates the accuracy of an acoustic guided wave technique in comparison to the traditional acoustic resonance and time-of-flight techniques and static bending tests.

Dynamic modulus of elasticity measurements were obtained of cylindrical rod samples using acoustic resonance, time of flight, and the new acoustic guided wave technique. The samples used for these measurements were 15 radiata pine (*Pinus radiata D.Don*) cylindrical rods. For comparison, measurements were also performed on five cylindrical rods each of Aluminium 6061-T6, Stainless Steel 304, and acetal (polyoxymethylene-copolymer). Static modulus of elasticity measurements were also performed for each of these samples using three-point bending tests to evaluate the accuracy of the three acoustic techniques.

The guided wave technique, which utilises the acoustic rod velocity, produced stiffness measurements similar to those of the resonance method. Both the resonance and guided wave techniques slightly overestimated the stiffness measurements compared to the static bending method for all samples except for aluminium. The ToF method produced stiffness measurements with overestimations that were almost double those of resonance and guided wave techniques.

The overestimations for the resonance and guided wave techniques were relatively small (0 to 2.5%) for aluminium and steel. It is possible that these overestimations may be due to errors in the static bending tests, such as calibration errors, rather than a systematic overestimation in the resonance and guided wave methods. Future work could be conducted to verify whether this is the case.

Acoustic resonance is considered in the literature to give stiffness measurements that are comparable to those of static bending tests. However, it cannot be used for standing trees as the technique requires two cut ends. The ToF technique can be used for this application, but it overestimates stiffness. The acoustic guided wave technique used in this work showed similar accuracy to resonance. Additionally, it would appear to have the potential, in theory, to be used on standing trees or seedlings (as well as logs and timber samples). It therefore has the potential to be used as an alternative to ToF for improved accuracy of stiffness measurements of trees and/or seedlings.

These measurements have been performed on relatively thin wooden rods with low moisture content. There would have been a limited number of guided wave modes present for the frequency range used. However, for larger-diameter rods, the wave propagation will be more complex. The larger the diameter, the more wave modes are expected to propagate for a given frequency. Additionally, standing trees and seedling will have a higher moisture content and bark on the surface. It is not clear what effect these factors will have on the accuracy of the guided wave technique.

Future work is intended to be performed using the guided wave technique on juvenile trees and seedlings with different diameters. Testing should also be performed on larger-diameter logs and standing trees. The effects of inhomogeneity, moisture content, temperature, grain angle variation, knots, and higher-order wave modes should be investigated to better understand the potential errors and uncertainties associated with the guided wave method.

## Figures and Tables

**Table 1:** Theoretical mechanical property values for aluminium, steel, and acetal used for dispersion curve generation. These values were mid-points of the values found online.

Material	Density (kg/m^3^)	Poisson’s Ratio	Young’s Modulus (GPa)
Aluminium [28]	2710	0.33	68.9
Steel [29]	8000	0.29	200.0
Acetal [30]	1410	0.43	2.7

**Table 2:** Measurement values for the fifteen wooden rod samples where $E_{L(res)}$, $E_{L(ToF)}$, and $E_{L(GW)}$ are the dynamic MoE values for the resonance, ToF, and guided wave methods, respectively, and $E_{S}$ are the static MoE values.

Sample	Density (kg/m^3^)	Acoustic Velocity (m/s)			$E_{L(\mathrm{res})}$ (GPa)	$E_{L(\mathrm{ToF})}$ (GPa)	$E_{L(\mathrm{GW})}$ (GPa)	$E_{S}$ (GPa)
		Resonance	ToF	GW				
1	504	5308	5184	5111	14.21	13.56	13.17	13.59
2	574	5621	5805	5653	18.12	19.33	18.34	17.00
3	484	5041	5281	5141	12.30	13.50	12.79	11.86
4	655	5308	5408	5357	18.45	19.16	18.80	18.73
5	517	5356	5592	5350	14.82	16.16	14.80	14.37
6	439	4468	4706	4487	8.77	9.73	8.84	8.23
7	468	4756	5166	5000	10.58	12.49	11.70	10.05
8	521	4917	5118	4984	12.59	13.64	12.94	10.93
9	639	5205	5408	5254	17.30	18.68	17.64	16.28
10	553	4935	4970	4804	13.48	13.67	12.76	12.32
11	395	4588	4598	4507	8.31	8.34	8.02	8.00
12	529	5092	5169	5052	13.71	14.13	13.50	13.01
13	545	5500	5493	5439	16.49	16.45	16.12	15.69
14	504	4684	4830	4753	11.07	11.77	11.39	9.99
15	468	4372	4276	4162	8.94	8.55	8.11	8.28

**Table 3:** Averaged measured MoE values for the wood, acetal, aluminium, and steel rod samples using resonance, time of flight, guided waves, and static bending tests.

Material	Density	$E_{L(\mathrm{res})}$	$E_{L(\mathrm{ToF})}$	$E_{L(\mathrm{gw})}$	$E_{S}$
	[kg/m^3^]	[GPa]	[GPa]	[GPa]	[GPa]
Wood	519 ± 68	13.26 ± 3.35	13.94 ± 3.54	13.26 ± 3.44	12.56 ± 3.37
Acetal	1380	2.94 ± 0.02	3.09 ± 0.03	2.95 ± 0.02	2.84 ± 0.07
Aluminium	2710	69.15 ± 0.0	70.33 ± 0.56	69.07 ± 0.0	69.30 ± 0.55
Steel	7809	211.45 ± 0.24	217.19 ± 3.68	211.3 ± 0.12	206.22 ± 1.03

**Table 4:** Pearson’s correlation coefficient between dynamic and static MoE values and density for the wooden samples.

	Density	$E_{L(\mathrm{res})}$	$E_{L(\mathrm{ToF})}$	$E_{L(\mathrm{GW})}$
$E_{L(res)}$	0.90	-	-	-
$E_{L(ToF)}$	0.89	0.97	-	-
$E_{L(GW)}$	0.90	0.98	0.99	-
$E_{S}$	0.89	0.99	0.96	0.97

**Table 5:** Percentage of overestimation relative to static bending tests of the dynamic MoE values, obtained using resonance, ToF, and guided waves.

Material	Resonance	ToF	Guided Waves
Wood	5.57%	11.6%	6.6%
Acetal	3.52%	8.7%	3.7%
Aluminium	−0.22%	1.5%	−0.3%
Steel	2.54%	5.3%	2.5%

## Data Availability

The original contributions presented in this study are included in the article.
